# Beyond Standard Defaults: How Reason Defaults Promote Green Product Purchase Through Reduced Psychological Reactance

**DOI:** 10.3390/bs16091655

**Published:** 2026-09-15

**Authors:** Wanda Ge, Yumei Jiang

**Affiliations:** Faculty of Economics and Management, Qilu University of Technology (Shandong Academy of Sciences), Jinan 250353, China; gewanda@qlu.edu.cn

**Keywords:** reason defaults, green product purchase, psychological reactance, green product typicality, nudging

## Abstract

Although default options have been increasingly applied to encourage sustainable consumption, their effectiveness in green product contexts remains constrained. Traditional default designs may unintentionally evoke psychological resistance when consumers perceive the recommended choice as insufficiently justified or as a threat to their autonomy. This study examines whether reason defaults can reduce such resistance and increase consumers’ stated green product choices. Across three empirical studies in hypothetical, lab-style online shopping contexts, the results show that reason defaults increase stated green product choices relative to standard defaults by reducing inferences of manipulative intent and compulsive feelings, with this effect stronger for typical green products and diminishing for atypical ones. This study extends reason defaults to green product purchase choice and offers practical implications for designing effective green nudging strategies.

## 1. Introduction

The transition toward sustainable consumption requires not only increasing consumers’ environmental awareness but also facilitating actual green purchasing behaviours. Despite growing public recognition of environmental issues, consumers’ intentions to choose green products frequently fail to translate into marketplace actions. This inconsistency reflects a persistent challenge in sustainable consumption: consumers often value environmental benefits while also weighing personal sacrifices associated with green purchases, such as higher prices, reduced convenience, or uncertainty about product performance (Frank & Brock, 2018; Arias Puentes & Trujillo, 2025). Therefore, it is critical to explore low-cost, effective nudging tools to bridge this gap in green product consumption. Nudging theory focuses on specific elements of individuals’ cognitive systems within decision environments. It aims to overcome cognitive and motivational limitations through low-cost, transparent interventions that do not rely on material incentives and preserve freedom of choice. These interventions can subsequently influence individuals’ existing behavioural patterns (Thaler & Sunstein, 2008; Hummel & Maedche, 2019; Fisher & Oppenheimer, 2025).

Default options are among the most prominent intervention tools in nudge theory. Their core mechanism involves preselecting a recommended choice and increasing the likelihood of selecting it by leveraging individuals’ status quo bias and cognitive inertia, while preserving freedom of choice (Ren & Liu, 2024). A substantial body of empirical research demonstrates that default options can produce significant nudging effects across various domains, including charitable donations, learning behaviours, and product consumption (Damgaard & Nielsen, 2018; Cadario & Chandon, 2020; Rauh et al., 2026).

However, the influence of defaults is not universal. In particular, their effectiveness may depend on how consumers interpret the intervention’s intent. When a recommended option is presented without sufficient explanation, individuals may question why that choice has been prioritised and perceive the intervention as an attempt to influence their decisions. Existing research indicates that default interventions often produce stronger effects in consumer decision-making settings, while their influence appears relatively limited in environmental domains (Jachimowicz et al., 2019; Sullivan et al., 2025). This discrepancy may stem from the limited transparency embedded in many default designs. When consumers cannot clearly understand why a particular option is preselected, they may interpret the intervention as an attempt to steer their decisions, which can evoke perceptions of reduced autonomy and subsequently activate psychological reactance (Buchanan et al., 2023). Beyond the characteristics of the default design itself, the underlying motivational orientation of the decision context may also determine the effectiveness of nudging strategies. Prior studies have demonstrated that nudges are generally more influential when they serve individuals’ personal interests than when they are intended to promote collective benefits (Yan & Yates, 2019). This concern is especially salient in green consumption because environmentally responsible choices often involve a trade-off between individual interests and collective benefits. Unlike purely utilitarian purchases, green products require consumers to consider not only personal outcomes but also broader environmental consequences (Baca & Reshidi, 2025). Therefore, minimizing threats to consumer autonomy while maintaining transparency and ease of opting out is critical for improving the effectiveness of default options (L. Wang et al., 2024).

Existing studies have primarily examined whether the presence of a default option influences decision outcomes. However, this perspective often overlooks the possibility that different forms of defaults may generate distinct psychological responses among decision makers (Cadario et al., 2026). For example, prior studies have classified defaults as opt-in or opt-out. In opt-in designs, individuals are not enrolled by default and must actively choose to participate. In opt-out designs, individuals are enrolled by default and must take action to withdraw (Kumar et al., 2014). These studies primarily examine how different preset choices influence decision outcomes. However, standard defaults assign the same option to all decision-makers and overlook individual differences in preferences. To address this limitation, personalized defaults have been proposed as a potential solution. Personalized defaults rely on predictive models to identify individuals’ optimal preferences or dynamically collect contextual information during the decision process to customise default settings (de Vries et al., 2025). Nevertheless, the effectiveness of personalized defaults depends heavily on accurate predictions of user preferences or on choice architects’ timely acquisition of high-quality data. Given practical constraints related to algorithmic costs and implementation complexity, personalized defaults still face substantial feasibility challenges (Jachimowicz et al., 2019).

Recent research on choice architecture has proposed reason defaults as an alternative design that balances behavioral guidance with individual autonomy (Desiraju & Dietvorst, 2023). Reason defaults retain the defining characteristic of conventional defaults by preselecting the option expected to be most beneficial for most decision-makers. At the same time, they explicitly explain why the selected option is designated as the default and when an alternative option may better serve an individual’s interests. The additional information is not intended merely to provide supplementary details about the available options; rather, it communicates the heterogeneity of individuals’ interests, clarifies the conditions under which different options may be more appropriate, and reduces the tendency to perceive the default option as having exclusive or authoritative status (Desiraju & Dietvorst, 2023). By incorporating preference heterogeneity into the design of default interventions, reason defaults provide consumers with a more transparent understanding of the decision environment. Such transparency may weaken consumers’ perceptions that their choices are being covertly influenced or controlled. Meanwhile, by presenting both the recommendation and the conditions under which other choices may be preferable, reason defaults allow consumers to retain greater autonomy and make decisions that are more consistent with their own preferences. Accordingly, we propose that reason defaults may outperform standard defaults in encouraging green product purchase choices. This advantage primarily stems from their ability to reduce consumers’ psychological reactance (Bruns & Perino, 2023; S. Wang, 2026).

Despite the emerging literature on reason defaults, several important questions remain insufficiently addressed. First, existing evidence has primarily examined reason defaults in relatively structured decision contexts, leaving their effectiveness in green consumption settings less well understood. Second, although prior research has demonstrated the potential advantages of reason defaults, the psychological processes through which they shape consumers’ responses have received limited empirical attention. Third, the boundary conditions that determine when reason defaults are more or less effective remain underexplored. To address these gaps, the present research examines whether reason defaults increase green product purchase choices relative to standard defaults, investigates inference of manipulative intent and compulsive feelings as underlying psychological mechanisms, and further examines green product typicality as a boundary condition of these effects. In doing so, this study extends research on default-based nudging by clarifying the role of reason defaults in green consumption and by integrating psychological reactance and product typicality into a unified explanatory framework.

## 2. Literature Review and Hypothesis Development

### 2.1. Reason Default

In behavioral decision research, defaults refer to pre-established choices that influence outcomes when individuals do not actively intervene in the decision process (Halpern et al., 2007). This concept was later introduced into behavioral decision research to describe situations in which a particular choice is automatically implemented when individuals fail to indicate their preferences.

A central explanation for default effects is that individuals frequently rely on the initial setting as a decision reference point, reducing the likelihood of switching to other available options. This phenomenon reflects status quo bias, whereby individuals tend to maintain existing conditions even when other options are available (Shevchenko et al., 2014). Prospect theory helps explain the persistence of default effects. Specifically, individuals tend to perceive the potential losses associated with abandoning the current option as more psychologically significant than the equivalent benefits that may result from switching choices. Consequently, decision-makers are often inclined to retain default options rather than incur the perceived risks of change (Steffen et al., 2020). Beyond loss aversion, defaults also influence behavior by simplifying the decision process. By eliminating the need to search for information, evaluate multiple alternatives, and actively execute a choice, default options reduce the cognitive effort required for decision-making. As a result, individuals may accept the preset option as a convenient strategy to minimize decision-related psychological costs and preserve the existing state through inaction (Reinders et al., 2024).

Despite the extensive application of default options in behavioral interventions, existing studies have not reached a consistent conclusion regarding whether defaults invariably produce positive default effects (Buchanan et al., 2023). Rather, empirical evidence suggests that default interventions can lead to divergent outcomes, including both “default effects” and “anti-default effects.” For instance, an online charitable giving study showed that default options could increase donations among certain individuals, leading them to contribute more than they originally intended. In contrast, the same intervention produced the opposite response among other participants, resulting in donations lower than their initial willingness (Altmann et al., 2019). The inconsistency of default effects may stem from several underlying factors. One possible explanation is that conventional default designs often assume that individuals have relatively homogeneous preferences. However, this assumption overlooks the diversity of consumers’ needs, values, and situational conditions, limiting defaults’ effectiveness in addressing individual differences. Another explanation concerns the limited informational support provided by traditional defaults. Many default interventions rely on simple recommendation cues to influence choices, which may obscure the rationale behind the recommended option and reduce consumers’ understanding of whether the preset choice is genuinely aligned with their personal interests (Paunov et al., 2019). Third, individuals are generally more receptive to pro-self defaults than pro-social defaults. Pro-social defaults primarily generate benefits for society rather than for individual decision-makers. Because individuals tend to pursue personal benefits and are less likely to engage in selfless behaviors, pro-social defaults may produce relatively weaker nudging effects (Everett et al., 2015).

To address the limitations of traditional default options, Desiraju and Dietvorst (2023) introduced the concept of reason default. Building on traditional defaults, reason defaults provide two additional pieces of information. First, they explain why a particular option is selected as the default. Second, they specify which groups of individuals may benefit from choosing alternative options under specific circumstances. Compared with traditional defaults, reason defaults not only set the option most beneficial for most decision-makers but also explain the rationale behind the default choice and clarify when alternative options may be more appropriate. This design acknowledges heterogeneity in individual preferences within decision contexts. It helps decision-makers understand when different options are suitable and reduces the tendency to see the default option as the only authoritative recommendation. Therefore, reason defaults represent an intermediate approach between traditional defaults and fully autonomous choice. By making subtle adjustments to information-based choice architecture, reason defaults maintain the efficiency of nudging interventions while providing decision-makers with more sufficient information for adaptive and personalized choices. Importantly, the defining feature of a reason default lies in how the rationale is incorporated into the choice architecture, rather than simply in the amount of information provided. Generic information about product attributes may improve consumers’ knowledge of the available alternatives, but it does not necessarily explain why a particular option has been preselected or indicate when an alternative may be more appropriate. In contrast, the information embedded in a reason default is directly connected to the default choice: it provides the rationale for the preselection. It identifies circumstances in which opting out may better fit an individual’s preferences. Thus, a reason default should be understood as a choice-architecture intervention that combines a preselected option with an explicit rationale and information about preference heterogeneity.

Desiraju and Dietvorst (2023), based on five experimental studies involving 4210 participants, found that this design can guide most individuals toward the default option while also helping individuals with different interests or minority preferences recognize when they should opt out. More importantly, their findings showed that reason defaults do not necessarily weaken the guiding effect of the default option itself. Rather, they can preserve the effectiveness of defaults while providing greater choice autonomy for individuals with minority interests. Therefore, reason defaults achieve a better balance between behavioral guidance and decision-making autonomy by avoiding the potential confusion associated with forced choices and mitigating the systematic biases that may arise when conventional defaults fail to accommodate heterogeneous preferences.

Although Desiraju and Dietvorst (2023) recently proposed the concept of reason default, its potential applications across different decision domains remain unclear. Previous studies have mainly focused on relatively structured decision environments, including retirement savings choices and academic testing contexts, where decision criteria are comparatively clear and well-defined. However, whether reason defaults can effectively influence consumer decisions involving environmentally responsible products remains largely unexplored. Addressing this gap, the present study extends the application of reason defaults to the context of green product consumption. Based on the design principles of reason defaults, we develop a reason-based default intervention and investigate its influence on consumers’ choices to purchase green products. In addition, this research examines psychological reactance as the psychological mechanism underlying the effectiveness of reason defaults. It identifies green product typicality as a boundary condition that determines when such interventions are more likely to succeed.

### 2.2. The Impact of Reason Defaults on Green Product Purchase

Green products differ from conventional alternatives in their environmental attributes, as they are designed to reduce ecological impacts while maintaining acceptable functional performance. These products typically improve resource efficiency, energy use, and environmental outcomes across different stages of the product life cycle (Sheng et al., 2019). However, developing green products requires additional investments in product design and green technology innovation. Moreover, using renewable and biodegradable materials, as well as efforts to reduce resource consumption, lower emissions, increase recycling rates, and improve resource utilization during production, increase production costs. Consequently, green products are often priced higher than conventional alternatives, requiring consumers to pay an environmental premium for their purchase (Kronrod et al., 2012). Due to these distinctive characteristics, purchasing green products often requires consumers to balance conflicts between personal and social benefits, as well as between immediate and long-term interests. As a result, consumers’ favorable attitudes toward green products are not always translated into actual purchasing behaviors, creating a persistent attitude–behavior gap in green consumption (Shehawy, 2023).

These trade-offs make reason defaults particularly relevant to green purchase decisions. A reason default retains the behavioral guidance provided by preselecting the green option while making the rationale for that preselection explicit. For example, consumers may be informed that “This product is recommended because it has a lower carbon footprint,” while also being told that “If you place greater emphasis on short-term economic benefits, conventional products are also available.” Importantly, the latter information is not included merely to present an additional product attribute or a disadvantage of the green product. Rather, it specifies the circumstances under which the alternative option may better serve consumers with different interests. Thus, compared with a standard default that primarily signals which option is recommended, a reason default gives consumers a clearer basis for understanding why the green option has been preselected and when opting out may be more consistent with their own preferences (Desiraju & Dietvorst, 2023).

In the context of green consumption, we argue that reason defaults are likely to outperform standard defaults in encouraging consumers to purchase green products. From the perspective of firms, developing green products not only contributes to environmental sustainability but also offers potential organizational benefits, including improved production efficiency, reduced energy and material use, and enhanced corporate reputation. Nevertheless, consumers often struggle to evaluate the authenticity of firms’ environmental commitments. In addition, choosing green products frequently requires consumers to balance environmental considerations against other attributes, such as price, convenience, and product performance. These challenges create considerable information asymmetry between firms and consumers in green product markets. Standard defaults typically direct consumers toward a predetermined green option. Although this approach can simplify decision-making and encourage environmentally responsible choices, it may also raise concerns about the intervention’s transparency. When consumers are presented with a recommended option without sufficient explanation of why it is preferable or when alternatives may be more suitable, they may perceive the intervention as an attempt to influence their decisions. Such perceptions can increase uncertainty and strengthen psychological resistance toward green product recommendations.

In contrast, by explaining the basis for the green default and acknowledging circumstances in which an alternative may better serve individual interests, reason defaults make the logic underlying the recommendation more transparent and explicitly accommodate preference heterogeneity. Consumers can therefore evaluate the recommended option in relation to their own preferences while retaining a stronger sense of control over the final decision. Consequently, reason defaults may reduce resistance to the default intervention and increase consumers’ willingness to choose the recommended green product. Therefore, we propose the following hypothesis:

**Hypothesis 1 (H1).**  *Compared with standard defaults, reason defaults are more effective in increasing consumers’ green product purchase choices.*

### 2.3. The Mediating Role of Psychological Reactance

Brehm’s (1966) psychological reactance theory explains individuals’ responses to situations in which they believe their ability to make independent choices has been limited. Under such circumstances, individuals may be motivated to protect their autonomy and resist influences perceived as restrictive (Rains, 2013). Psychological reactance is not merely a negative emotional response. Rather, it represents a motivational mechanism that drives attitude change and behavioral responses. When external interventions are perceived as threats to personal autonomy, individuals may not comply with the recommended behaviors. Instead, they may activate defensive psychological responses and develop attitudes or behavioral tendencies that oppose the intended actions. Through such responses, individuals attempt to reestablish their freedom of choice. In consumer research, proactive product recommendations and manipulative advertising messages are often perceived as potential threats to decision-making freedom (Ha et al., 2025). Accordingly, psychological reactance theory has been widely applied to explain consumers’ responses to various marketing interventions. For example, aggressive salesperson recommendations, persuasive advertising tactics, and unsolicited product recommendations may trigger psychological reactance when consumers perceive them as attempts to constrain their choices. In actual purchase situations, consumers exposed to unsolicited information may not only reject the recommendations but also choose behaviors opposite to the suggested ones (Yang & Xu, 2025).

Psychological reactance typically involves two key psychological processes: inference of manipulative intent and compulsive feeling (H. He et al., 2018; Pierre et al., 2026). Inference of manipulative intent refers to individuals’ tendency to infer the underlying motives of information providers or choice architects when encountering information designed to influence their behavior (Dai et al., 2025). When individuals perceive such intentions as manipulative or deceptive, they are more likely to experience a stronger sense of threatened freedom, which subsequently triggers psychological reactance. Compulsive feeling represents an immediate emotional response that occurs when individuals perceive threats to their freedom of choice. It integrates cognitive evaluations of restricted autonomy with negative emotional arousal, manifesting as feelings such as irritation, anger, and a sense of behavioral constraint (Hu & Wise, 2021; Liu et al., 2020). These two psychological processes can occur simultaneously and reinforce each other. Inference of manipulative intent provides the cognitive foundation for compulsive feeling, whereas compulsive feeling further strengthens negative attributions regarding manipulative intentions.

As noted earlier, standard defaults primarily function by assigning a predetermined option, while offering little information regarding the rationale behind the recommendation. This limited transparency may lead consumers to question the underlying motives of choice architects and interpret the default arrangement as a strategy driven by firms’ interests rather than as an effort to facilitate consumer decision-making. Such perceptions can increase concerns about external influence and reduce consumers’ acceptance of the recommended choice. By comparison, reason defaults do not merely provide additional information; rather, they explicitly link the rationale to the preselected option by explaining why it has been designated as the default and under what circumstances an alternative may better serve individual interests. This structure makes the basis and boundaries of the recommendation more explicit and acknowledges that the default option is not necessarily optimal for every consumer. As a result, consumers may be less likely to interpret the preselection as an attempt by the choice architect to covertly steer them toward a predetermined outcome, thereby reducing their inference of manipulative intent. Reason defaults may also reduce compulsive feelings by altering consumers’ perceptions of the intervention’s restrictiveness. By explicitly indicating when opting out may be appropriate, reason defaults signal that choosing an alternative is a legitimate and acceptable course of action rather than a deviation from the recommended choice. This acknowledgment of preference heterogeneity allows consumers to evaluate the default in relation to their own interests and maintain a stronger sense of control over the final decision. Thus, although the option remains preselected, the intervention is less likely to be perceived as constraining consumers’ freedom of choice, thereby alleviating feelings of pressure and compulsion. Based on the above arguments, we propose the following hypotheses:

**Hypothesis 2 (H2).**  *Compared with standard defaults, reason defaults reduce consumers’ inference of manipulative intent.*

**Hypothesis 3 (H3).**  *Compared with standard defaults, reason defaults reduce consumers’ compulsive feelings.*

Once psychological reactance is activated, individuals tend to adopt various strategies to restore their threatened freedom of choice. One of the most common responses is to engage in behaviors opposite to the recommended actions (J. He et al., 2026). According to the persuasion knowledge model, consumers actively activate internal cognitive structures regarding persuaders’ motives and their own coping strategies when exposed to persuasive information (Isaac & Grayson, 2017). When consumers recognize explicit persuasive intentions and further infer that such intentions are manipulative, they activate defensive mechanisms. Consequently, they become less receptive to the recommended products or information (Eisend & Tarrahi, 2022). Meanwhile, compulsive feelings resulting from restricted choice freedom represent an intense negative emotional experience that directly motivates oppositional behaviors. Prior research has consistently shown that psychological reactance reduces consumers’ favorable attitudes toward advertisements and products and negatively influences purchase decisions (Ju & Jun, 2025). In the relationship between default options and green product purchases, when consumers attribute green product defaults to firms’ profit-seeking motives or regulatory compliance rather than genuine environmental responsibility, they may become skeptical of environmental claims and develop negative evaluations of corporate integrity (Taube & Vetter, 2019). Moreover, strong compulsive feelings may intensify resistance reactions, leading consumers to choose conventional products to reaffirm their autonomy. Consequently, psychological reactance may deteriorate consumers’ attitudes toward green products and reduce their purchase choices.

According to psychological reactance theory, individuals’ cognitive evaluations and emotional responses toward choice architectures represent critical mechanisms underlying their behavioural responses. Psychological reactance can function as a mediating variable with both antecedents and consequences. Its bridging role between external environmental information and individual behaviours has been widely examined in contexts such as targeted advertising, personalized recommendations, and health communication (Ahn & Ham, 2022). As an external intervention cue, standard defaults may trigger consumers’ inference of manipulative intent and compulsive feelings because their opaque design can be attributed to firms’ self-serving motives. In contrast, reason defaults provide explicit explanations for the recommended option and clarify the conditions under which consumers may select alternative options. By enhancing transparency and perceived legitimacy, reason defaults preserve consumers’ sense of autonomy, thereby reducing inference of manipulative intent and alleviating compulsive feelings. Therefore, compared with standard defaults, reason defaults are expected to reduce consumers’ inferences of manipulative intent and compulsive feelings, thereby enhancing their green product purchase choices. Based on the above arguments, we propose the following hypotheses:

**Hypothesis 4 (H4).**  *Inference of manipulative intent negatively influences green product purchase choice and mediates the relationship between default options and green product purchase choice.*

**Hypothesis 5 (H5).**  *Compulsive feeling negatively influences green product purchase choice and mediates the relationship between default options and green product purchase choice.*

### 2.4. The Moderated Mediating Role of Green Product Typicality

Green product typicality refers to the extent to which a product matches consumers’ cognitive category of “green products” (Usrey et al., 2020; Zheng et al., 2026). According to prototype theory, consumers possess cognitive prototypes for specific product categories that represent the most salient features of typical category members (Rosch & Mervis, 1975). For typical green products, green attributes constitute the core functions and essential identity of the product. These products have strong and salient associations with consumers’ cognitive prototypes of “green products,” such as electric vehicles, biodegradable plastic bags, and energy-efficient household appliances (Y. He et al., 2026). In contrast, the green attributes of atypical green products are often secondary or supplementary features rather than essential elements that define product identity. These products are relatively distant from consumers’ cognitive prototypes of “green products,” such as recycled-material running shoes, energy-efficient high-performance laptops, and conventional foods using environmentally friendly packaging (Yao et al., 2025).

Green product typicality may therefore determine how consumers interpret the rationale embedded in a reason default. When a product is highly typical of the green-product category, a rationale emphasizing environmental benefits is consistent with consumers’ existing category expectations. This consistency makes the basis for preselecting the green option easier to understand and more congruent with consumers’ existing beliefs about why the product should be recommended. Consequently, compared with a standard default, a reason default is more likely to reduce consumers’ inference that the preselection represents an attempt to manipulate their decisions. Moreover, because the reason default explicitly acknowledges when an alternative may better serve individual interests, consumers are more likely to perceive that opting out remains a legitimate choice, thereby reducing compulsive feelings and preserving a greater sense of control over the decision.

When a product is atypical of the green-product category, however, the environmental rationale underlying the default is less consistent with consumers’ existing category expectations. Consumers may therefore find the basis for preselecting the green option less immediately convincing or relevant to their product evaluation. Under these conditions, explicitly providing a rationale may be less effective in changing consumers’ interpretation of the default intervention. As a result, the differences between reason defaults and standard defaults in the inference of manipulative intent and compulsive feelings are expected to become weaker, thereby diminishing the indirect effect of reason defaults on green product purchase choice through psychological reactance. Accordingly, we propose the following hypotheses:

**Hypothesis 6 (H6).**  *Green product typicality moderates the mediating effects of inference of manipulative intent and compulsive feelings in the relationship between default options and green product purchase choice.*

**Hypothesis 6a (H6a).**  *For typical green products, compared with standard defaults, reason defaults evoke lower levels of inference of manipulative intent and compulsive feelings. Inference of manipulative intent and compulsive feelings mediate the relationship between default options and green product purchase choice.*

**Hypothesis 6b (H6b).**  *For atypical green products, there are no significant differences between standard defaults and reason defaults in terms of inference of manipulative intent and compulsive feelings. Consequently, the mediating effects of inference of manipulative intent and compulsive feelings become insignificant.*

**Figure 1:**
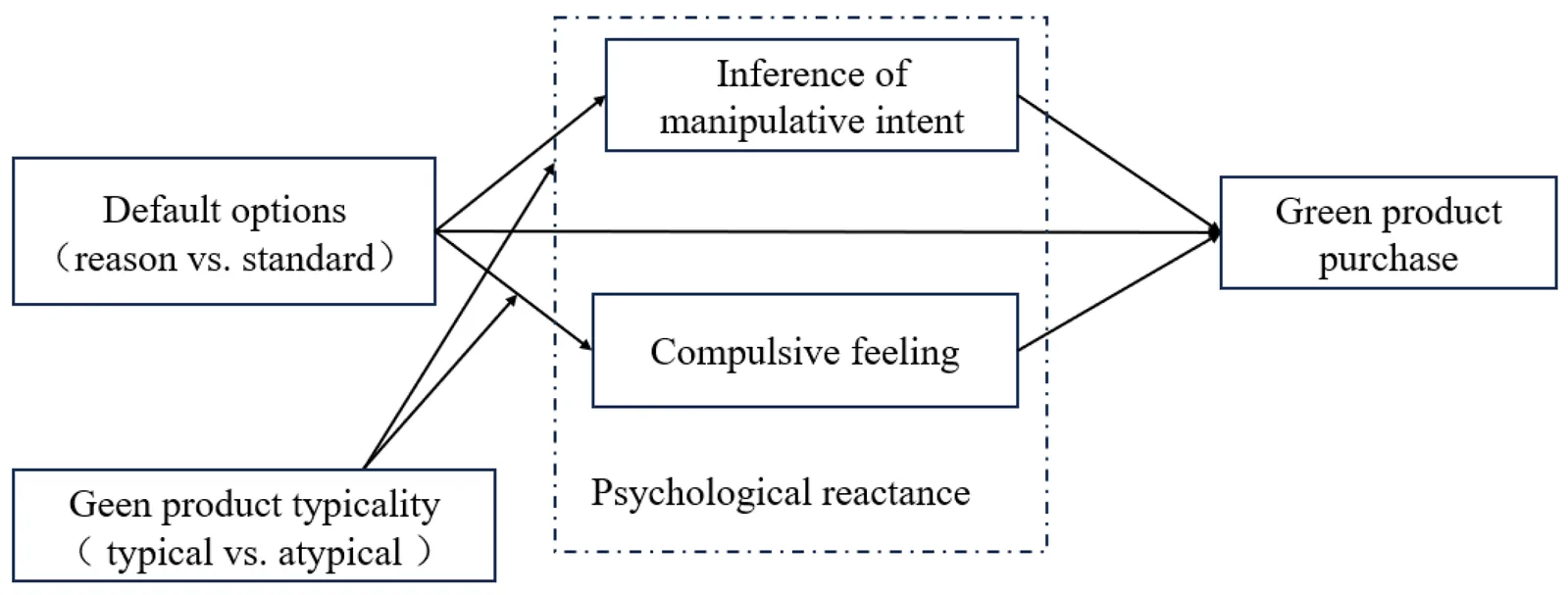
The conceptual model.

Figure 1 presents the conceptual framework developed in this study.

## 3. Study 1: Main Impact of Reason Defaults on Green Product Purchase

The purpose of Study 1 was to assess whether the design of default options affects consumers’ green product purchase choices. By comparing reason defaults with standard defaults, we examined whether providing explanatory information alongside default recommendations could generate stronger purchase choices toward green products.

### 3.1. Pretest

Before the main experiments, we conducted a pretest to select a suitable green product stimulus. The product needed broad consumer relevance and high everyday usage, ensuring participants could evaluate the stimulus with comparable familiarity. This approach helped minimize experimental bias associated with differences in product knowledge, experience, and category perceptions. Specifically, we aimed to identify a green product category that consumers perceived as neither highly typical nor atypical.

To identify an appropriate green product stimulus, we first conducted consumer interviews. During the interviews, participants received a brief explanation of green products and were then asked to recall green products they commonly encountered in daily life or had previously purchased. We analyzed and categorized the responses, identifying seven frequently mentioned green product categories: laundry detergent, toilet paper, washing machines, automobiles, electric kettles, stationery, and thermos cups. Subsequently, we recruited 50 participants through the Wenjuanxing online survey platform. We measured green product typicality using a 7-point scale anchored by “atypical green product” (1) and “typical green product” (7). The results of the one-sample *t*-test showed that participants’ typicality ratings for laundry detergent (*M* = 4.07) were close to the midpoint of the scale (4), *t*(49) = 0.553, *p* = 0.583. This indicates that participants did not perceive either product as particularly representative or unrepresentative of green products. Therefore, laundry detergent was selected as the stimulus product for Studies 1 and 2.

The research team developed two fictitious laundry detergent products from the same brand and informed participants that they could choose between a “green product” and a “regular product.” Following the stimulus design used by Griskevicius et al. (2010), participants were told the two laundry detergents were identical in capacity, cleaning effectiveness, and other functional attributes. The only difference was that the green product would not cause any environmental pollution. Considering that green products typically involve at least a 20% price premium compared with conventional products (Lin & Chen, 2016), we set the price of the regular laundry detergent at RMB 18 per bottle and the price of the green laundry detergent at RMB 22 per bottle. To ensure the effectiveness of the product type manipulation, we recruited 50 undergraduate students who did not participate in the main experiment. Participants were randomly assigned to evaluate one of the two products’ environmental friendliness using a 7-point scale (1 = extremely environmentally unfriendly, 7 = extremely environmentally friendly). The results showed that the green product (*M* = 5.84, SD = 0.79) was perceived as significantly more environmentally friendly than the regular product (*M* = 2.92, SD = 0.80; *t* = 18.29, *p* < 0.001). Moreover, the mean ratings of both products significantly differed from the scale midpoint (4), indicating that the manipulation of product type was successful.

### 3.2. Sample

Before data collection, the required sample size for adequate power was calculated using G*Power 3.1 software. Based on the methodological guidelines provided by (Brock, 2023), the significance level (α) was set at 0.05, power at 0.8, and the effect size at 0.3, df at 2. A minimum sample size of 108 valid participants was required for the study. In total, 163 individuals were recruited. Before proceeding with the main experiment, participants’ familiarity with the target product category was assessed. Specifically, they rated their agreement with the statement, “I am a frequent buyer of laundry detergent when I run out of it,” on a 7-point Likert scale ranging from 1 (“strongly disagree”) to 7 (“strongly agree”). Participants who selected 6 (“agree”) or 7 (“strongly agree”) were considered sufficiently familiar with the product category and were retained for subsequent analyses. We excluded five participants who reported insufficient familiarity (scores below 6). Following this screening procedure, the final sample consisted of 158 participants. Among the participants, 53.7% were female, 72.8% were aged between 26 and 40 years, 58.3% held a bachelor’s degree, and 43.0% reported a monthly income between RMB 5000 and 8000.

### 3.3. Procedures

Study 1 adopted a between-subjects design in which default type was manipulated across three conditions: reason default, standard default, and control. After completing the experimental design, data collection was carried out on the Wenjuanxing online data platform. To ensure experimental control, participants were randomly allocated to one of the three conditions with equal probability. The platform conducted quality screening by detecting duplicate responses, checking IP information, and applying additional criteria to improve data reliability. Participants who completed the study received RMB 2 as compensation.

Before the experiment, participants’ environmental attitudes were measured to ensure no significant differences across the three groups, confirming the effectiveness of random assignment (*F* = 0.134, *p* > 0.05). Accordingly, environmental attitude was not included as a covariate in the main analyses. After entering the experiment, participants read the instructions and were told they were browsing an online shopping website to purchase laundry detergent. The survey system then randomly assigned them to one of three conditions: the control condition, the standard default condition, or the reason default condition. Participants subsequently chose between the green and regular products. In the control condition, neither option was preselected. After reading the product information, participants chose between the two laundry detergents. Under the standard default condition, the green product’s purchase option was preselected. Participants were informed that they could choose between the two options and could change the default selection if desired. In the reason default condition, the green product option was preselected. Additionally, a notification box was provided below the option, stating: “We recommend the green laundry detergent because it helps protect the environment. However, if environmental benefits are not a priority, the regular laundry detergent is also an appropriate choice.” Participants could click the notification box to view this information and close it after reading. Participants’ access to the reason information was optional rather than mandatory. Because the platform did not retain participant-level click records for the notification box, we were unable to verify whether all participants assigned to the reason-default condition actually viewed the reason information. Finally, participants were asked to choose between the green product and the regular product. The experimental materials are presented in Appendix A. The valid sample sizes in the reason default group, standard default group, and control group were 54, 52, and 52 participants, respectively.

### 3.4. Measures

Environmental attitude was measured using four adapted items from Milfont and Duckitt (2010) (Cronbach’s α = 0.87; 1 = strongly disagree, 7 = strongly agree). The items were: “I actively participate in environmental protection activities,” “Humans are seriously damaging the environment,” “I make efforts to conserve natural resources,” and “To achieve sustainable development, governments should regulate the exploitation of natural resources.”

### 3.5. Results

To assess whether default options influence consumers’ green product choices, purchase decisions were coded as a binary variable, with conventional product choices coded as 0 and green product choices coded as 1. A chi-square analysis evaluated differences in green product selection among the three experimental conditions.

The analysis revealed significant variation in green product choice rates across conditions (χ^2^ = 24.451, *p* < 0.001). Specifically, green product adoption was highest in the reason default condition (90.7%), followed by the standard default condition (71.4%), and the control condition (47.3%). Subsequent pairwise comparisons indicated that participants exposed to reason defaults selected green products significantly more often than those exposed to standard defaults (χ^2^ = 6.369, *p* < 0.05) and the control condition (χ^2^ = 23.987, *p* < 0.001). Moreover, the standard default condition also resulted in a higher proportion of green product choices than the control condition (χ^2^ = 6.325, *p* < 0.05). Overall, these results suggest that incorporating default interventions can effectively encourage green product choices, with reason defaults generating a stronger behavioral effect than standard defaults. Therefore, H1 was supported.

### 3.6. Discussion

The findings of Study 1 demonstrate that default options can effectively increase consumers’ willingness to purchase green products, and that reason defaults produce a stronger effect than standard defaults. Compared with standard defaults, reason defaults provide consumers with explicit rationales underlying the preset option, thereby enhancing decision transparency and perceived information credibility. The results indicate that reason defaults have a stronger effect on consumers’ green product purchase choices than standard defaults. These findings suggest the potential importance of examining the psychological processes underlying the effectiveness of reason defaults. Accordingly, Study 2 extends the investigation by exploring whether perceived manipulative intent and feelings of being compelled mediate how default types influence consumers’ choices to purchase green products.

## 4. Study 2: Mediating Role of Psychological Reactance

Study 2 was designed to further examine the differential effects of standard defaults and reason defaults on consumers’ green product purchase choice and to investigate the mediating roles of inferences about manipulative intent and compulsive feelings. Specifically, we examined whether reason defaults enhance green product purchase choice by reducing consumers’ inference of manipulative intent and compulsive feelings.

### 4.1. Sample

Before the formal experiment, we calculated the required sample size for adequate power using G*Power. Following (Brock, 2023) methodology, the significance level (α) was set at 0.05, power at 0.8, and the effect size at 0.3, df at 1. The calculation determined a minimum requirement of 88 valid responses. Before the experiment began, all participants were informed that the study was conducted solely for academic purposes and that their data would be treated anonymously and confidentially. During data screening, responses from participants who did not complete the entire questionnaire were excluded. The final valid sample comprised 136 participants. Among the participants, 52.2% were female, 67.6% were aged between 26 and 40 years, 52.2% held a bachelor’s degree, and 41.1% reported a monthly income between RMB 5000 and 8000.

### 4.2. Procedures

Study 2 employed a single-factor (default type: reason default vs. standard default) between-subjects experimental design. Data were collected online through Wenjuanxing, following the same recruitment and collection procedures as Study 1. Before the experiment, participants’ environmental attitudes were measured. The results showed no significant difference between the two groups (*F* = 0.320, *p* > 0.05), indicating that the random assignment was successful. Participants were informed that they would complete a simulated online shopping task. After making their purchase choice, they completed measures of inference of manipulative intent and compulsive feeling. The valid sample sizes in both the reason default group and the standard default group were 68 participants.

### 4.3. Measures

Inference of manipulative intent was measured using four adapted items from Lwin and Phau (2014) (Cronbach’s α = 0.92). Although these items were originally developed in the context of advertising, they capture the extent to which consumers perceive that an external agent attempts to influence or manipulate their decision-making process, which is conceptually consistent with perceived manipulative intent in the context of default options. We made minor wording modifications to fit the default-choice context in the present study. Participants rated the extent to which they agreed with the following statements: “The purchase option setting in the previous task was intended to influence my choice,” “The purchase option setting in the previous task was intended to manipulate my behavior,” “The purchase option setting in the previous task was designed to achieve its own goals rather than help me make the optimal decision,” and “The purchase option setting in the previous task was unusual.”

Compulsive feeling was measured using four adapted items from Edwards et al. (2002) (Cronbach’s α = 0.90). These items, though originally used in studies on pop-up advertising, tap into the affective and cognitive responses associated with threats to freedom and perceived coercion. We treat these items as a proxy measure of consumers’ compulsive feelings in the default option context. Participants indicated their agreement with the following statements: “The purchase option setting in the previous task forced me to make a choice,” “The purchase option setting in the previous task prevented me from concentrating on other information,” “The purchase option setting in the previous task led me to abandon the most appropriate choice,” and “The purchase option setting in the previous task interfered with my decision-making.”

### 4.4. Results

#### 4.4.1. Direct Effect Analysis

To examine the effect of default options on green product purchase decisions, this study used a chi-square test to compare green product choices between the two groups. The results revealed a significant difference in green product choice rates between the two groups (χ^2^ = 9.256, *p* < 0.01). Specifically, 89.7% of participants in the reason-default condition chose the green product, compared with 66.1% in the standard-default condition. These results indicate that, compared with standard defaults, reason defaults are more effective in increasing consumers’ willingness to purchase green products. Therefore, H1 was further supported.

#### 4.4.2. Mediation Analysis

We first assessed whether psychological reactance differed across the two default conditions. We compared participants exposed to reason defaults with those exposed to standard defaults. Participants exposed to reason defaults perceived significantly less manipulative intent (*M* = 3.02, SD = 1.65) compared with those exposed to standard defaults (*M* = 3.84, SD = 1.75, *t*(134) = 2.763, *p* < 0.05). A similar pattern emerged for compulsive feelings, with participants in the reason default condition reporting lower levels of this response (*M* = 2.72, SD = 1.55) than those in the standard default condition (*M* = 3.73, SD = 1.73, *t*(134) = 3.547, *p* < 0.05). Together, these results offered preliminary evidence in favor of H2 and H3.

Second, we conducted a parallel mediation analysis using the bootstrapping method with 5000 resamples (Hayes, 2017; Model 4). Default option was coded as the independent variable (0 = standard default, 1 = reason default), with the inference of manipulative intent and compulsive feeling entered as mediators and green product purchase choice as the dependent variable. The correlation between the two mediators was significant (r = 0.860, *p* < 0.001), supporting their inclusion in a parallel model. Table 1 presents the results.

As shown in Table 1, the default option had a significant negative effect on inference of manipulative intent (*b* = −0.82, SE = 0.29, *p* < 0.05) and compulsive feeling (*b* = −1.01, SE = 0.28, *p* < 0.05). Therefore, H2 and H3 were supported.

Furthermore, when both mediators were entered simultaneously as predictors of purchase choice, inference of manipulative intent (*b* = −1.28, SE = 0.49, *p* < 0.05) and compulsive feeling (*b* = −1.04, SE = 0.38, *p* < 0.05) each significantly and negatively predicted purchase choice.

The total indirect effect was significant (Effect = 2.10, SE = 9.77, 95% BootCI [0.7956, 5.8593]). More importantly, the specific indirect effect via inference of manipulative intent (Effect = 1.05, SE = 4.04, 95% BootCI [0.2935, 3.0016]) and the specific indirect effect via compulsive feeling (Effect = 1.05, SE = 6.41, 95% BootCI [0.2254, 3.3170]) were both significant, as their bootstrap confidence intervals did not include zero. These results indicate that both perceived manipulative intent and psychological reactance mediate the effect of the default option on green product purchase choice. Therefore, H4 and H5 were supported.

### 4.5. Discussion

Standard defaults, due to their lack of explanatory information, are more likely to trigger consumers’ inference of manipulative intent and compulsive feelings, thereby reducing purchase choice. In contrast, reason defaults provide rationales for the preset choice, thereby reducing psychological reactance and subsequently enhancing green product purchase choice. Given the high correlation between inference of manipulative intent and compulsive feeling (r = 0.860), these two pathways substantially overlap and should therefore be interpreted as related proxies for psychological reactance rather than fully independent mechanisms. Building on the findings of Study 2, Study 3 further investigates the moderating role of green product typicality in the relationship between default type and psychological reactance. It also examines whether green product typicality moderates the indirect effects of default type on green product purchase choice through psychological reactance.

## 5. Study 3: Moderated Mediating Role of Green Product Typicality

Study 3 aims to examine the moderating role of green product typicality in the relationship between default type and psychological reactance. Furthermore, it investigates the moderated mediation effect of green product typicality on the indirect relationship between default type and green product purchase choice through psychological reactance.

### 5.1. Pretest

The purpose of the pretest was to select appropriate typical and atypical green products. Based on the studies of Usrey et al. (2020) and Huang and Sengupta (2020), we selected an energy-efficient desk lamp as the typical green product and an environmentally friendly mouse as the atypical green product. The environmental attributes of the energy-efficient desk lamp are primarily reflected in core characteristics such as energy conservation and reduced carbon emissions, which are highly consistent with consumers’ typical perceptions of green products. In contrast, the environmental attributes of the environmentally friendly mouse are primarily reflected in peripheral characteristics, such as the materials used and the production process, which are relatively less consistent with consumers’ typical perceptions of green products.

To ensure the effectiveness of the product typicality manipulation, we recruited 100 university students who did not participate in the main experiments and randomly assigned them to either the energy-efficient desk lamp condition or the environmentally friendly mouse condition, with 50 participants in each condition. Participants were asked to evaluate the perceived green product typicality of the assigned product. Green product typicality was measured using three items: “This product fits my typical impression of a green product,” “This product is a representative green product,” and “I believe this product has typical green attributes.” All items were measured using a 7-point Likert scale ranging from 1 (“strongly disagree”) to 7 (“strongly agree”). An independent-samples *t*-test showed that participants in the energy-efficient desk lamp condition reported significantly higher perceived green product typicality (*M* = 5.77, SD = 0.83) than those in the environmentally friendly mouse condition (*M* = 2.88, SD = 0.71), *t*(98) = 19.210, *p* < 0.001. These results indicate that the manipulation of green product typicality was successful, supporting the use of the energy-efficient desk lamp and environmentally friendly mouse as the typical and atypical green product stimuli, respectively, in the subsequent experiments.

### 5.2. Sample

Based on G*Power sample size calculation (α = 0.05, 1 − β = 0.8, effect size = 0.25), the minimum required sample size was determined as 128 participants. Before the experiment, all participants provided informed consent. We recruited 300 participants, who received monetary compensation for their participation. After data screening and excluding participants who failed the attention check, the final sample comprised 278 valid responses. Among them, 52.1% were female, 66.5% were aged between 26 and 40 years, 60.9% held a bachelor’s degree, and 42.5% reported a monthly income between RMB 5000 and 8000. Participants were randomly assigned to one of the four experimental conditions. Participants’ environmental attitudes were measured before the experiment to ensure no significant differences across conditions. The results showed that participants’ environmental attitudes did not differ significantly among the four groups (*F* = 0.635, *p* > 0.05), indicating that the random assignment was effective.

### 5.3. Procedures

This study employed a 2 (default type: reason default vs. standard default) × 2 (green product typicality: typical vs. atypical) between-subjects experimental design. The data were collected through the Wenjuanxing platform after the experimental design was completed. Participants were randomly assigned to the four experimental groups with equal probability. At the beginning of the experiment, participants read the experimental instructions and were informed that they were browsing an online shopping website. Participants were then randomly assigned by the survey system to one of four experimental conditions: Group A (reason default/typical green product), Group B (standard default/typical green product), Group C (reason default/atypical green product), or Group D (standard default/atypical green product). The default-option manipulation was identical to that used in Study 1. Participants in Groups A and B were asked to make a purchase choice between an energy-efficient desk lamp and a conventional desk lamp, whereas participants in Groups C and D were asked to choose between an environmentally friendly mouse and a conventional mouse. The energy-efficient desk lamp and environmentally friendly mouse were priced at RMB 60, whereas the conventional desk lamp and conventional mouse were priced at RMB 50.

Participants then completed measures of inference of manipulative intent and compulsive feeling. The measurement items were identical to those used in Study 2 and were assessed using 7-point Likert scales. The experimental materials are presented in Appendix B. The final valid sample sizes were 71 participants in Group A, 70 in Group B, 69 in Group C, and 68 in Group D.

### 5.4. Results

#### 5.4.1. Impact of Default Options and Green Product Typicality on Green Product Choice

The choice to purchase a conventional product was coded as 0, whereas the choice to purchase a green product was coded as 1.

**Figure 2:**
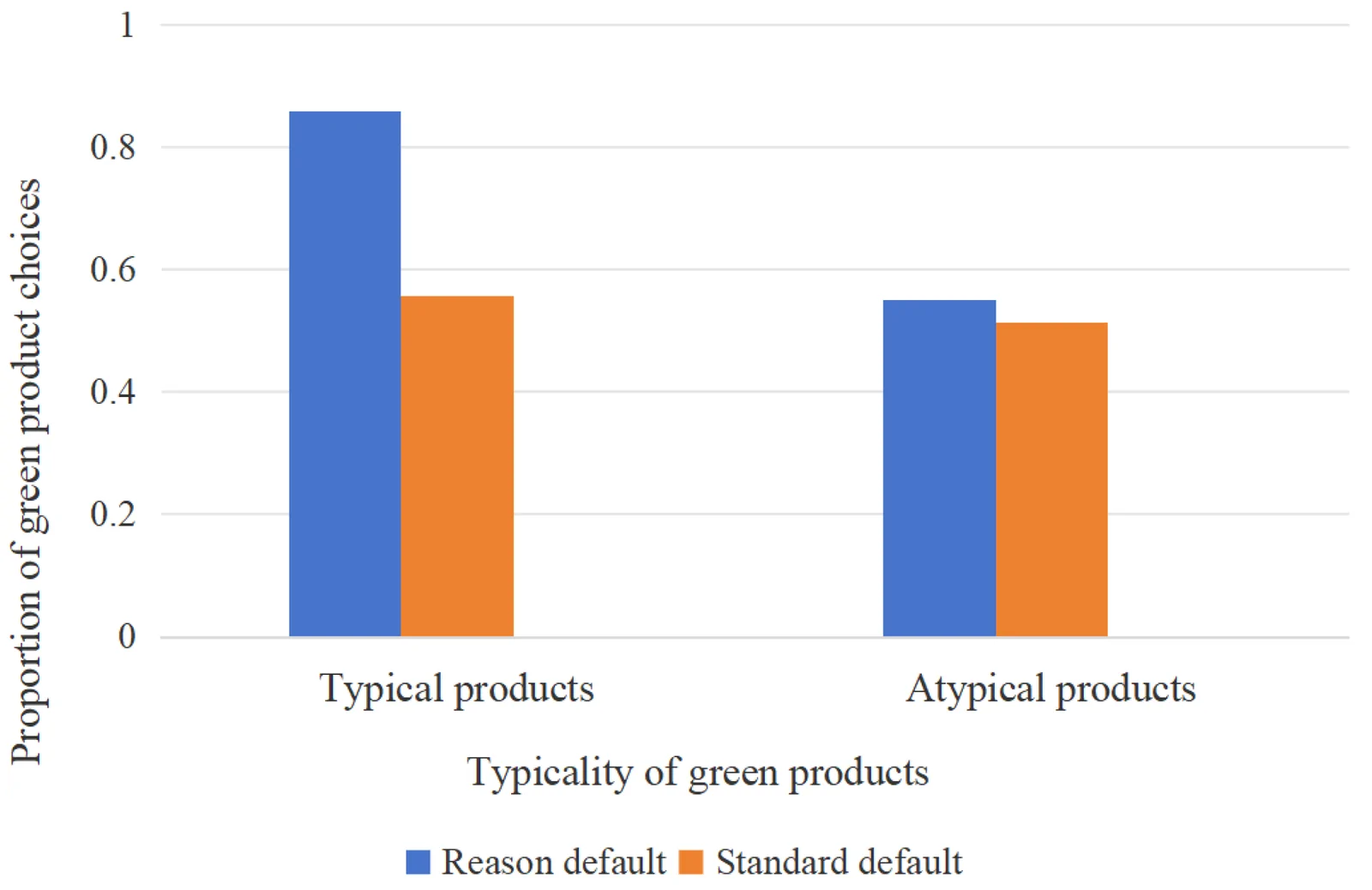
The impact of default options and green product typicality on green product purchase choice.

The numbers of participants who chose the green product in Groups A, B, C, and D were 61, 39, 38, and 35, respectively, corresponding to 85.9%, 55.7%, 55.1%, and 51.4%, respectively, as shown in Figure 2. A chi-square test revealed a significant difference in green product choice across the four groups (χ^2^ = 23.064, *p* < 0.001). Specifically, the difference between Groups A and B was significant (χ^2^ = 15.590, *p* < 0.001), whereas the difference between Groups C and D was not significant (χ^2^ = 0.250, *p* = 0.617).

A two-way ANOVA was conducted to examine the effects of green product typicality and default options on green product choice. The main effect of green product typicality was significant, *F*(1, 274) = 9.778, *p* = 0.002. The main effect of default options was also significant, *F*(1, 274) = 9.076, *p* = 0.003. Moreover, the interaction effect between green product typicality and default options was significant, *F*(1, 274) = 5.620, *p* = 0.018.

#### 5.4.2. Impact of Default Options and Green Product Typicality on Psychological Reactance

To test the moderated mediation hypothesis, a moderated mediation analysis was conducted using PROCESS Model 7, with default option as the independent variable; inference of manipulative intent and compulsive feeling of being compelled as indicators of psychological reactance; green product typicality as the moderator; and green product purchase choice as the dependent variable. The analysis was based on 5000 bootstrap samples—Table 2 and Table 3 present the results. Table 2 presents the moderating effect of green product typicality on the relationship between default option and psychological reactance. Table 3 presents the moderating effect of green product typicality on the mediating effect of psychological reactance.

As shown in Table 2, the interaction between default option and green product typicality had a significant effect on inference of manipulative intent (*b* = −1.12, SE = 0.40, *t* = −2.79, *p* < 0.05, 95% CI [−1.9130, −0.3322]). Simple slope analyses showed that, under the atypical green product condition, the default option did not significantly affect inference of manipulative intent (*b* = 0.32, SE = 0.28, *t* = 1.12, *p* > 0.05, 95% CI [−0.2407, 0.8852]). In contrast, under the typical green product condition, the default option significantly reduced consumers’ inference of manipulative intent (*b* = −0.80, SE = 0.28, *t* = −2.84, *p* < 0.05, 95% CI [−1.3552, −0.2455]). These findings indicate that the default option effectively reduces consumers’ perceptions of the inference of manipulative intent when the green product is typical.

The interaction between default option and green product typicality also significantly affected compulsive feeling (*b* = −1.11, SE = 0.38, *t* = −2.93, *p* < 0.05, 95% CI [−1.8608, −0.3637]). Further simple slope analyses showed that, under the atypical green product condition, the default option did not significantly affect compulsive feeling (*b* = 0.34, SE = 0.27, *t* = 1.25, *p* > 0.05, 95% CI [−0.1959, 0.8703]). However, under the typical green product condition, the default option significantly reduced consumers’ compulsive feeling (*b* = −0.77, SE = 0.27, *t* = −2.90, *p* < 0.05, 95% CI [−1.3006, −0.2496]). Overall, green product typicality significantly moderated the effects of the default option on both psychological reactance responses. Specifically, the effects of the default option in reducing inference of manipulative intent and compulsive feeling primarily emerged in the typical green product condition.

#### 5.4.3. Moderated Mediation Analysis

As shown in Table 3, the moderated mediation effect of inference of manipulative intent was significant (95% CI [0.3710, 2.6247]). Specifically, for atypical green products, the indirect effect of inference of manipulative intent was not significant (95% CI [−1.1691, 0.3251]), whereas this mediating effect was significant for typical green products (95% CI [0.2961, 1.8513]). These results indicate that inference of manipulative intent mediated the relationship between default option and green product purchase choice for typical green products, but not for atypical green products.

Similarly, green product typicality moderated the mediating effect of compulsive feeling in the relationship between default type and green product purchase choice (95% CI [0.4768, 3.0433]). Specifically, for atypical green products, the indirect effect of compulsive feeling was not significant (95% CI [−1.3855, 0.3333]), whereas the mediating effect was significant for typical green products (95% CI [0.3352, 2.1420]). These findings suggest that compulsive feeling mediated the effect of default option on green product purchase choice for typical green products, but not for atypical green products. Therefore, H6a, H6b, and H6 were supported.

### 5.5. Discussion

The findings of this study demonstrate that green product typicality significantly moderates the mediating mechanism through which default type influences purchase choice via psychological reactance. Specifically, for typical green products, reason defaults effectively reduce consumers’ compulsive feelings and inference of manipulative intent, thereby increasing green product purchase choice. Psychological reactance serves as the underlying mediating mechanism in this process. However, for atypical green products, reason and standard defaults do not differ significantly in terms of consumers’ psychological reactance, and the mediating effects of psychological reactance are not significant. These findings reveal the boundary conditions of default-based nudging effects. When green products have high category typicality, the additional rational information provided by reason defaults can effectively alleviate consumers’ concerns about the intervention’s legitimacy and reduce psychological reactance. In contrast, when green attributes are ambiguous or atypical, consumers find it more difficult to establish a direct association between the default option and environmental value. Consequently, the explanatory power of rational information is weakened, and the psychological advantage of reason defaults diminishes.

## 6. Conclusions

### 6.1. Theoretical Contributions

Building on prior research on reason defaults (Desiraju & Dietvorst, 2023), this study extends the application of reason defaults to green product purchase choice. We reveal the underlying mechanism through which default options operate in the context of green product purchase and identify an important boundary condition of their effectiveness. Across three studies, we find that, compared with standard defaults, reason defaults can increase consumers’ green product choices by reducing psychological reactance. In addition, green product typicality is identified as an important boundary condition of this effect.

First, this study extends research on reason defaults to the context of green product purchase choice. Prior research (Desiraju & Dietvorst, 2023) has shown that, in general choice contexts, reason defaults can help decision-makers with heterogeneous interests make choices that better serve their own interests. Specifically, reason defaults can guide most individuals toward the default option while helping a minority with different interests choose an alternative option. However, in green product purchase decisions, consumers may perceive green product recommendations as an influence strategy intended to encourage socially desirable behavior, making default interventions more likely to trigger psychological reactance. Our findings show that, compared with standard defaults, reason defaults can also effectively increase consumers’ green product choices in this context. More importantly, while guiding most consumers toward the default option, reason defaults also provide a more autonomous choice space for the minority of consumers sensitive to default interventions, without sacrificing the default option’s guiding effect on the majority.

Second, drawing on psychological reactance theory, this study reveals the underlying psychological mechanism through which reason defaults operate in the context of green consumption. In Desiraju and Dietvorst’s (2023) research, the advantages of reason defaults were attributed to enhanced transparency, reduced decision difficulty, and improved understanding of the options. Our study shows that, in the context of green consumption, reason defaults alleviate psychological reactance by reducing consumers’ inference of manipulative intent and compulsive feeling, thereby increasing their willingness to choose green products. Prior research has not examined this mechanism. By doing so, this study extends the theoretical explanation of default effects beyond traditional perspectives of status quo bias and cognitive inertia to a psychological reactance framework. It demonstrates the applicability of this framework in the context of green consumption.

Third, this study further identifies green product typicality as an important boundary condition determining the effectiveness of reason defaults. Our findings show that, for typical green products, consumers’ perceptions of green attributes are relatively well established, making the rationale provided by reason defaults easier to integrate into the decision-making process and thereby more effective in reducing psychological reactance. In contrast, for atypical green products, even when a rational explanation is provided, the recommendation may be less consistent with consumers’ existing product perceptions and expectations. As a result, consumers may still interpret such information as a more subtle form of manipulation, thereby weakening or even offsetting the advantages of reason defaults. Identifying this moderating effect adds a product-category-level boundary condition to reason default theory. It suggests that default strategies should account for consumers’ existing knowledge and perceptions of the product category.

### 6.2. Managerial Implications

This research provides clear managerial implications for firms and policymakers seeking to promote green product consumption through default-based interventions. The findings demonstrate that not all default options generate equivalent nudging effects. Reason defaults can significantly reduce consumers’ psychological reactance, thereby more effectively enhancing green product purchase choice. Moreover, this effect is particularly pronounced for typical green products.

First, the effectiveness of green defaults depends not only on which option is preselected but also on how the recommendation is communicated. Firms should therefore avoid implementing green defaults as simple forced selections and instead accompany these recommendations with clear explanations regarding their underlying rationale. In particular, consumers should be informed about why a specific green option may be beneficial and when alternative choices may better fit their individual needs. For example, online retailers may recommend environmentally friendly laundry detergents or biodegradable tableware as default options while simultaneously presenting information about their environmental advantages and acknowledging cases in which other products may be more suitable. This transparent and balanced approach enables firms to maintain the behavioral benefits of default interventions while minimizing consumers’ concerns about manipulation, strengthening perceived credibility, and improving acceptance of green alternatives.

Second, the applicability of reason defaults should be considered in relation to the characteristics of the green product itself. Our findings suggest that product typicality influences how strongly consumers respond positively to reason default interventions. For products with highly recognizable environmental attributes, reason defaults are particularly effective because consumers can more easily connect the recommendation with existing environmental beliefs. Accordingly, firms may prioritize reason defaults in such product categories. However, when promoting less typical green products, relying solely on default strategies may be insufficient. In these situations, firms should provide additional information that helps consumers recognize the product’s environmental value and establish stronger associations between the product and sustainability. Combining reason defaults with other behavioral interventions, such as social norm appeals or feedback mechanisms, may further enhance consumers’ engagement with atypical green products.

Third, firms and policymakers should incorporate consumer autonomy and transparency as central considerations when developing green nudging strategies. The present research highlights psychological reactance as an important factor that can undermine the effectiveness of default interventions. Thus, choice architectures should be designed to reduce perceptions of coercion and avoid communication practices that may be interpreted as manipulative. Providing objective, evidence-based, and balanced information, along with accessible opt-out opportunities, can help consumers maintain a sense of control over their decisions. Such practices not only improve the ethical acceptability of green nudges but also contribute to their sustained effectiveness over time.

### 6.3. Limitations and Future Research

Although this research provides experimental evidence for the effectiveness of reason defaults in promoting green product purchase choice and identifies the mediating role of psychological reactance, several limitations remain that warrant further investigation and extension.

First, effective intervention strategies for atypical green products require further investigation. This study finds that reason defaults fail to significantly enhance green purchase choice in the context of atypical green products. Future research could explore combined intervention strategies by conducting experimental studies that integrate reason defaults with other persuasive signals. For example, a hybrid strategy combining reason defaults with third-party certification labels could be applied to products such as recycled-material apparel and energy-efficient electronic devices. Such research could systematically examine which signals are most effective in rebuilding consumers’ trust in green attributes, reducing psychological reactance, and ultimately restoring the effectiveness of default-based interventions.

Second, this study employed a limited set of product categories, including laundry detergent, an energy-saving desk lamp, and an eco-friendly mouse. It did not systematically examine high-involvement or high-priced green products. Consumer decision-making processes for low-involvement products are relatively straightforward, whereas high-involvement products typically involve more complex attribute trade-offs. Therefore, whether the explanatory information provided by reason defaults can effectively reduce psychological reactance and enhance purchase choice in high-involvement consumption contexts remains an open question. Future research could extend the investigation to a broader range of green products and compare the effectiveness of reason defaults across products with different levels of consumer involvement.

Third, the treatment fidelity of the reason-default manipulation could be further improved. In the reason-default condition, participants were instructed to click the notification box to view the rationale for the preselected option, but this action could not be made mandatory. Because the platform did not retain participant-level click records, we were unable to verify whether all participants assigned to the reason-default condition actually viewed the reason information. This optional exposure limits the interpretation of the reason-default effect to some extent. Future research could require participants to view the reason information before making their choices or record their actual exposure to the information to ensure greater treatment fidelity.

Fourth, the generalizability of the findings is constrained by the sampling context. Participants in the present research were recruited in China through Wenjuanxing, an online research platform. Although the platform facilitated standardized implementation of the experimental procedures, reliance on participants from a single country and a single online panel may limit the generalizability of the findings to other cultural and institutional contexts and broader consumer populations. Cultural differences in environmental values, responses to behavioral interventions, and perceptions of decision autonomy may shape consumers’ responses to reason defaults. Future research should therefore replicate the present findings using samples from different countries, cultural contexts, and recruitment sources.

Finally, the present studies relied on hypothetical, unpaid purchase choices in simulated online shopping scenarios rather than actual transactions involving real economic consequences. Although scenario-based experiments provide greater experimental control over the choice environment, consumers may respond differently when their decisions require actual monetary expenditure and result in real product acquisition. Accordingly, the present findings should be interpreted as evidence regarding stated green product choices in experimental settings rather than as direct evidence of actual marketplace purchasing behavior. Future research could employ incentive-compatible experiments, field experiments, or actual transaction data to examine whether the effects of reason defaults persist when consumers face real financial consequences.

## Figures and Tables

**Table 1:** Mediation effect of inference of manipulative intent and compulsive feeling.

Effect	Path	Coef.	SE	t/Z	*p*	LLCI	ULCI
Direct effect	Default option → Inference of manipulative intent	−0.82	0.29	−2.7626	0.0065	−1.4067	−0.2329
	Default option → Compulsive feeling	−1.01	0.28	−3.5475	0.0005	−1.5739	−0.4471
	Inference of manipulative intent → Green product purchase choice	−1.28	0.49	−2.6155	0.0089	−2.2538	−0.3229
	Compulsive feeling → Green product purchase choice	−1.04	0.38	−2.6751	0.0075	−1.8051	−0.2785
Indirect effect	Total indirect effect	2.10	9.7720	—	—	0.7956	5.8593
	Inference of manipulative intent	1.05	4.0488	—	—	0.2935	3.0016
	Compulsive feeling	1.05	6.4156	—	—	0.2254	3.3170

**Table 2:** Moderating effect of green product typicality on the effect of default option on psychological reactance.

Variable	Predictor	b	SE	t	*p*	LLCI	ULCI
Inference of manipulative intent	Default option	0.3223	0.2859	1.1270	0.2607	−0.2407	0.8851
	Green product typicality	0.3103	0.2849	1.0890	0.2771	−0.2506	0.8712
	Interaction	−1.1226	0.4015	−2.7960	0.0055	−1.9130	−0.3322
	Simple slope analysis: atypical green products	0.3223	0.2859	1.1270	0.2607	−0.2407	0.8852
	Simple slope analysis: typical green products	−0.8004	0.2819	−2.8395	0.0049	−1.3552	−0.2455
Compulsive feeling	Default option	0.3372	0.2708	1.2451	0.2141	−0.1959	0.8703
	Green product typicality	0.2968	0.2698	1.1001	0.2722	−0.2344	0.8281
	Interaction	−1.1123	0.3802	−2.9252	0.0037	−1.8608	−0.3637
	Simple slope analysis: atypical green products	0.3372	0.2708	1.2451	0.2141	−0.1959	0.8703
	Simple slope analysis: typical green products	−0.7751	0.2669	−2.9038	0.0040	−1.3006	−0.2496

**Table 3:** Moderated mediation of reactance.

Mediator	Green Product Typicality	Effect	SE	LLCI	ULCI
Inference of manipulative intent	Atypical	−0.3752	0.3811	−1.1691	0.3251
	Typical	0.9318	0.4120	0.2961	1.8513
Index of moderated mediation		1.3070	0.5905	0.3710	2.6247
Compulsive feeling	Atypical	−0.4525	0.4340	−1.3855	0.3333
	Typical	1.0403	0.4612	0.3352	2.1420
Index of moderated mediation		1.4928	0.6647	0.4768	3.0433

## Data Availability

The data presented in this study are available on request from the corresponding author. The data are not publicly available due to privacy and ethical restrictions.

## Appendix A. The Experimental Materials in Study 1

A.

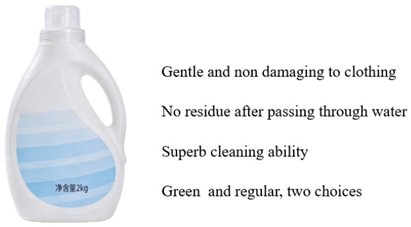

B.

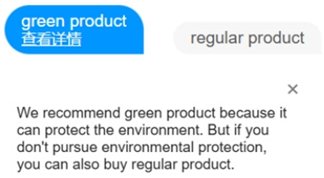

C.

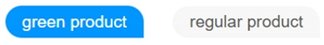

D.

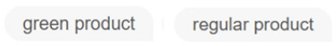

Note. A: example of the presentation of products (laundry detergent). B–D: examples of options participants could choose from, respectively, from the reason default, standard default, and control conditions. The Chinese term “净含量 2 kg” is Net weight 2 kg; The Chinese term “查看详情” is a standard interface button meaning “View Details”.

## Appendix B. The Experimental Materials in Study 3

A.

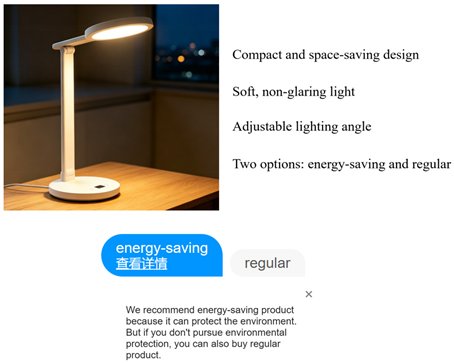

B.

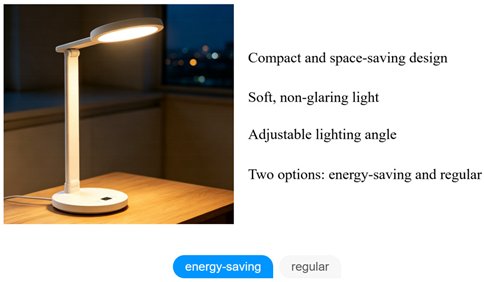

C.

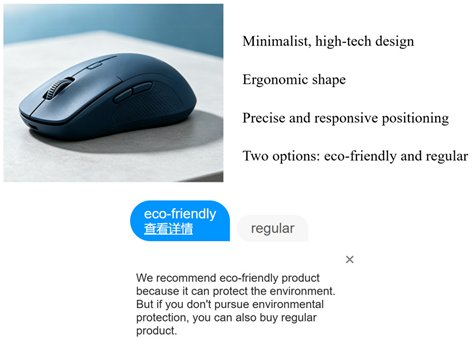

D.

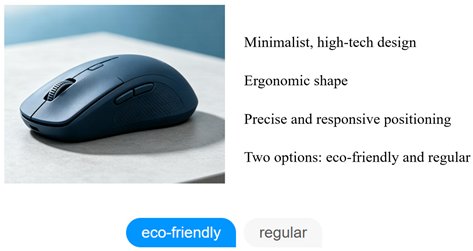

Note. A: rational default under a typical green product. B: Standard default under typical green product; C: rational default under atypical green product context. D: Standard default under atypical green product. The Chinese term “查看详情” is a standard interface button meaning “View Details”.
